# Prediction of antigenic epitopes on protein surfaces by consensus scoring

**DOI:** 10.1186/1471-2105-10-302

**Published:** 2009-09-22

**Authors:** Shide Liang, Dandan Zheng, Chi Zhang, Martin Zacharias

**Affiliations:** 1 School of Engineering and Science, Jacobs University Bremen, Campus Ring 1, D-28759 Bremen, Germany; 2 Department of Radiation Oncology, Massey Cancer Center, Virginia Commonwealth University, Richmond, VA, 23298, USA; 3 The Center for Plant Science Innovation, School of Biological Sciences, University of Nebraska, Lincoln, NE, 68588, USA; 4 Physics Department, Technical University Munich, James Franck Str. 1, D-85747 Garching, Germany

## Abstract

**Background:**

Prediction of antigenic epitopes on protein surfaces is important for vaccine design. Most existing epitope prediction methods focus on protein sequences to predict continuous epitopes linear in sequence. Only a few structure-based epitope prediction algorithms are available and they have not yet shown satisfying performance.

**Results:**

We present a new antigen Epitope Prediction method, which uses ConsEnsus Scoring (EPCES) from six different scoring functions - residue epitope propensity, conservation score, side-chain energy score, contact number, surface planarity score, and secondary structure composition. Applied to unbounded antigen structures from an independent test set, EPCES was able to predict antigenic eptitopes with 47.8% sensitivity, 69.5% specificity and an AUC value of 0.632. The performance of the method is statistically similar to other published methods. The AUC value of EPCES is slightly higher compared to the best results of existing algorithms by about 0.034.

**Conclusion:**

Our work shows consensus scoring of multiple features has a better performance than any single term. The successful prediction is also due to the new score of residue epitope propensity based on atomic solvent accessibility.

## Background

Realistic prediction of protein surface regions that are preferentially recognized by antibodies (antigenic epitopes) can help in the design of vaccine components and immuno-diagnostic reagents. Antigenic epitopes are classified as continuous or discontinues epitopes. If the residues involved in an epitope are contiguous in the polypeptide chain, this epitope is called a continuous epitope or a linear epitope. On the other hand, a discontinuous or non-linear epitope is composed of residues that are not necessarily continuous in the polypeptide sequence but have spatial proximity on the surface of a protein structure. A significant fraction of epitopes are discontinuous in the sense that antibody binding is not fully determined by a linear peptide segment but also influenced by adjacent surface regions [1].

However, the majority of available epitope prediction methods focus on continuous epitopes due to the convenience of the investigation in which the amino acid sequence of a protein is taken as the input. Such prediction methods are based upon the amino acid properties including hydrophilicity [2,3], solvent accessibility [4], secondary structure [5], flexibility [6], and antigenicity [7]. In addition, based on the known linear epitope databases such as Bcipep [8] and FIMM [9], there also exist some methods using machine learning algorithms such as Hidden Markov Model (HMM) [10], Artificial Neural Network (ANN) [11], and Support Vector Machine (SVM) [12,13] to locate linear epitopes. A study by Blythe and Flower has demonstrated that, using single-scale amino acid property profiles, a linear epitope prediction method was not able to predict epitope location reliably [14], whereas Greenbaum et al. showed that, using a combination of more than one amino aid property scale, machine learning algorithms could improve prediction accuracy[15].

Unlike linear epitope prediction, only a small number of studies have been performed so far on the prediction of discontinuous epitopes employing structural information of a target protein. Although such studies are of highly importance, the small number of available structures of antibody-antigen complexes limits this kind of studies. Several databases, such as IEDB [16], SACS [17], and CED [18], collected all existing structures of antibody-antigen complexes from the PDB bank. With the 3-dimentional structures of proteins as input, a few methods have been designed to predict putative antigenic epitopes by using conservation score, amino acid statistics, accessibility, and spatial information [19-24]. Ponomarenko and Bourne evaluated DiscoTope[20] and CEP[19] along with six other protein binding site prediction methods by benchmarking on 62 epitope structures and 82 antibody-antigen structures. They concluded that none of those prediction methods have a performance exceeding 40% precision and 46% recall[25]. Clearly, there is still a large gap between the strong need for antigenic epitope prediction and the low accuracy that existing prediction methods can achieve.

Use of multiple features could potentially improve performances on predicting antigenic epitopes, but this raises another question: are the properties effective for the limited number of antigens with available complex structures also work as well for all antigens? In this study, we tested 6 properties, which were used in protein/antibody binding site prediction previously, with the published databases plus the most recently released PDB entries. We found that the performances of the 6 terms were quite different for the two databases. Nevertheless, consensus prediction of the 6 terms resulted in reasonable accuracy for both databases.

## Methods

### Protein datasets

#### Protein Dataset 1

48 antigen-antibody complexes with resolution <3.0 Å were selected from the 59 representative antigen-antibody complexes compiled by Ponomarenko and Bourne[25]: 2ADF, 1FE8, 1BGX, 1E6J, 1EGJ, 1FSK, 1H0D, 1IQD, 1JRH, 1LK3, 1MHP, 1NL0, 1NSN, 1OAZ, 1ORS, 1PKQ, 1RJL, 1SY6, 1TZI, 1WEJ, 1YJD, 1YY9, 1ZTX, 2JEL, 1A14, 1NCA, 1BVK, 1JHL, 1NDG, 1P2C, 1JPS, 1AR1, 1EO8, 1QFU, 1EZV, 1OSP, 1FJ1, 1FNS, 1G9M, 1R3J, 1N8Z, 1NFD, 1TQB, 2VDL, 1V7M, 1XIW, 2AEP, and 1R0A. All entries were released before January 2006 except for 2VDL, which was the new version of original entry 1TXV. This dataset was used to derive residue epitope propensities.

#### Protein Dataset 2

22 antigen-antibody complexes and their unbound structures were selected from protein docking Benchmark 2.0 [26]. Benchmark 2.0 was published in 2005 and overlaps with Protein Dataset 1. The complex structures in this dataset were used to locate the antibody binding sites. Interface residues on the surface of unbound antigens were used to optimize the parameters for the binding site prediction method and considered as the training set.

#### Protein Dataset 3

This dataset was curated by us and served as an independent test set, which has 17 antigen-antibody complexes released between February 2006 and October 2008. Within this window, there were 180 entries returned by querying the PDB with a resolution <3.0 Å, using key words "antibody" and "complex". All complexes of antibodies with non-protein-ligands were manually removed from those 180 structures. Subsequently we performed a sequence alignment for antigens in the remaining complexes and Protein Dataset 1. A complex was kept if the maximum sequence identity between its antigen and any antigen in Protein Dataset 1 was less than 35% in local alignment. For a complex with a maximum sequence identity in the range of 35~50%, we accepted the complexes if the binding topology was not the same as the corresponding complex in Protein Dataset 1. The same criterion was also applied on any two complexes within Protein Dataset 3 itself. As a result, a total of 17 antigen-antibody complexes were selected. The unbound structures of the antigens in those 17 complexes were also obtained from the PDB. The structure with the best resolution was selected if there was more than one protein structure available in PDB. For the case that an antigen's unbound structure was not available, its bound structure in a complex with another protein was used for evaluation.

### Definition of surface residues, surface patches, and interface residues

Following previous work [27], we consider an amino acid residue as a surface residue if the relative accessibility of its side chain is greater than 6% with probe radius = 1.2Å. Also to confirm with previous work on protein binding site prediction [27] a surface patch is defined as a central surface residue and its 19 nearest surface neighbors in space. Solvent vector constraints [28] were applied in order to avoid patches sampled on different sides of a protein surface. An interface residue is the surface residue with solvent accessibility decreased more than 1 Å^2 ^upon association.

### Six terms for antibody binding site prediction

Residue epitope propensity[29], conservation score[29], side chain energy score[29], contact number[20], surface planarity score[30], and secondary structure composition[5] were exploited for antibody binding site prediction. We previously used the first three terms for protein-protein interface prediction (PINUP[29]). In an independent comparative study the PINUP method showed the highest prediction accuracy compared to other published interface prediction approaches [31]. The last three terms have already been used for antibody binding site prediction by others. We describe the details of those six terms in the following paragraphs.

#### Residue epitope propensity

The score of antibody binding site propensity, *E*_propensity_(*i*), is defined as

(1)

Where  and  are the contribution of residue type *r *to the antibody binding site and to the protein surface area, respectively, *S*_r _and  are the relative accessible surface area of residue *r *at the sequence position *i *and the average relative accessible surface area of surface residues of type *r*, respectively. The C_α _atom of Gly is considered as a side chain atom for convenience. Since antigen-antibody interfaces have different residue composition compared with other protein-protein interfaces, we used Protein Dataset 1 to derive residue antibody binding site propensity instead of using the former residue interface propensity score[29]. Here,  and  were obtained from statistical analysis of Protein Dataset 1. Some antigens in Dataset 1 have multiple epitopes. Those residues belonging to any of the epitopes were considered as antibody-binding interface residues. The values of  for 20 amino acid residues were obtained from the statistical analyses on 41 antigens in Protein Dataset 1.

#### Residue conservation score

Residue conservation was measured by the self-substitution score from the sequence profile. Sequence profiles were obtained by three rounds of PSI-BLAST searches with the BLOSUM62[32] substitution matrix. The conservation score at the position *i *is defined as

(2)

where *M*_*ir *_is the self-substitution score in the position-specific substitution matrix generated from PSIBLAST for the residue type *r *at sequence position *i*, and *B*_*rr *_is the diagonal element of BLOSUM62 for residue type *r*. Usually, protein-protein interface residues are more conserved than other surface residues due to functional constraints, and hence, the conserved surface residues in the unbound structure will be predicted as interface residues. The residues in the antibody-binding site, however, are less conserved than other surface residues due to the constraint of the host immune system. The unconserved residues are considered as the putative antibody binding site residues.

#### Side-chain energy score

The exact expression for side chain energy score can be found in Eq. (3) in PINUP [29]. It was calculated from the side-chain energies of all possible rotamers for a given residue type at a sequence position whereas other sequence positions have native residue types and observed atomic coordinates. The weights of the energy function were optimized so that the native residue was predicted energetically favorable at each position of the training proteins[33] The assumption is that the residues at the antibody binding site may have a higher energy score than other surface residues so that the free energy of the antigen-antibody system could go down significantly upon association.

#### Contact number

The residue contact number is the number of C_α _atoms in the antigen within a distance of 10 Å of the C_α _atom of residue *i *[20]. A residue with a small contact number was considered as an antibody binding site residue.

#### Planarity score

The planarity of each surface patch was calculated by evaluating the root mean squared (rms) deviation of all the C_α _atoms in the surface patch from the least squares plane through the atoms. The rms deviations were inverted such that a high planarity score for a patch was interpreted as a planar patch and antibody binding site[34].

#### Secondary structure composition

This score was defined as the fraction of patch residues forming turns or loops in all 20 patch residues. Following Chou & Fasman's method[35], the α-helix and β-sheet were defined as four or more consecutive residues having ϕ, ψ angles within 40° of (-60°, -50°) and three or more residues having ϕ, ψ angles within 40° of (-120°, 110°) or (-140°, 135°), respectively. The remaining regions were considered turns and loops.

### Prediction of discontinuous epitopes

#### Prediction with one term

Given the structure of an antigen, all of its surface residues were sampled, and hence all its corresponding surface patches could be obtained. The score of a patch for one scoring function is given by the average value of its scores for all 20 residues. Based on a certain threshold, the central residue of the top percentile patch was predicted as an interface residue. In case of secondary structure composition and contact number score if a patch was not ranked above the threshold but scored the same as any top ranked patch, the patch was also added into the top-ranked patch set.

#### Prediction with consensus scoring

To take the advantage of the multiple features, we used a voting mechanism with the above described six scoring functions. A patch was considered as an interface patch if five of the all six terms scored it into the top-ranked patch set. We did not use the vote mechanism of all six votes from the six scoring function because one surface patch with a small contact number could not have a high planarity score at the same time. The number of predicted residues with each single term is the same but the threshold of how many top ranked patches shall be kept can be varied to yield predictions with different sensitivities.

### Patch score derived by unevenly averaged single-residue scores

Patch analysis is widely used in protein binding site prediction. In general, it is assumed that each residue in the patch contributes equally to the patch score. Here, we exploited patch scoring with a weight decreasing with the distance from the center of the patch,

(3)

where *E*_residue_(*k*) is the score of residue *k *in the patch; *d *is the distance between residue *k *and the central residue of the patch; *T *is the parameter to be optimized during training.

### Evaluation methods

Sensitivity and precision were defined as the ratios of the number of correctly predicted interface residues to the number of real interface residues and to the number of all predicted interface residues, respectively. Specificity is defined as the fraction of correctly predicted surface residues in the total number of observed surface residues. As recommended by Ponomarenko & Bourne[25], the area under the receiver operating characteristic curve (AUC) was used as the primary evaluation metric. A receiver operating characteristic (ROC) curve represents a dependency of sensitivity and (1-specificity). To obtain the ROC curve, we increased the number of predicted residues (or the predicted residue with the single term in consensus prediction) in steps of 1% of total surface residues. A java program downloaded from , was used to calculate the AUC.

## Results and Discussions

### Predictions with one term

As a first test, we used each single term described in the Materials and Methods section to evaluate Protein Dataset 2 (training set) and Protein Dataset 3 (testing set). The residue antibody binding site propensity was independently derived from Protein Dataset 1 (Table 1). The correlation coefficient between the antibody binding site propensity and the protein-protein interface propensity values used in a previous study [29] is -0.15. This indicates clearly that antigen-antibody complexes have unique interface properties and that propensity value specifically derived from antigen-antibody complexes should be used for antibody binding site prediction. Especially, cysteine has a exceptionally large negative value of  because it is seldom found at antibody binding sites [20] and usually has a small solvent accessibility. On the other hand, cysteine is enriched relative to protein surfaces in general at other protein-protein interfaces[29] Excluding cystine, the correlation coefficient between the antibody binding site propensity and the protein-protein interface propensity rises to 0.3.

**Table 1:** The values of  for twenty amino acid residues.

***Amino Acid***		***Amino Acid***	
Ala	-0.392	Leu	-1.31
Arg	0.316	Lys	0.021
Asn	0.446	Met	1.06
Asp	-0.307	Phe	0.979
Cys	-7.36	Pro	0.017
Gln	-0.006	Trp	-0.07
Glu	-0.492	Val	-0.826
Gly	0.463	Ser	-0.004
His	0.207	Thr	-0.062
Ile	0.334	Tyr	0.979

is the contribution of residue type *r *to the area of antibody binding site;  is the contribution of residue type *r *to the protein surface area;  is the average relative accessible surface area of surface residues of type *r*.

Antibody binding site propensity alone results in a quite accurate prediction (AUC = 0.637) for the training set (Protein Dataset 2). This is because the training set overlaps with Protein Dataset 1, which was used to derive the antibody binding site propensity values for the 20 amino acids. The prediction accuracy is considerably lower (0.577) for the testing set. It is possible that the propensity values were over trained. Other terms showed prediction accuracies only slightly better than random. Most of the contributing terms showed quite different performance for the training and testing sets (Table 2). For example, the score based on secondary structure composition showed no prediction ability for the training set, whereas it was the most effective term for the testing set. Antibody binding site propensity, conservation score, and contact number were the three most effective terms for the training set but were only medium predictors for the testing set.

**Table 2:** AUC values for training and testing datasets predicted by the single term

***Evaluation terms***	***Training set*^*a*^**	***Testing set*^*b*^**
Binding site propensity	0.637	0.577
Conservation score	0.593	0.564
Side chain energy score	0.555	0.569
Contact number	0.59	0.556
Planarity score	0.53	0.554
Fraction of turns & loops	0.489	0.587

^a ^Antigen-antibody complexes from protein docking benchmark 2.0. ^b ^17 recently released antigen-antibody complex structures in PDB. Unbound structures of both databsets were used for prediction and bound structures were used for identification of interface residues. The AUC values were calculated and averaged for all the proteins in two datasets, respectively.

### Prediction results for the training dataset with consensus scoring

**Figure 1:**
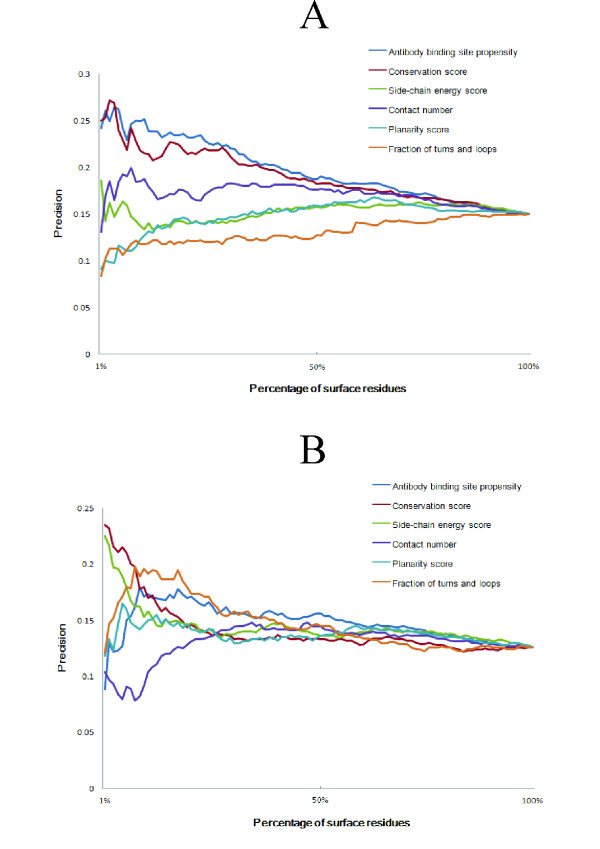
**Correlation between precision and the number of predicted residues (a) Training set; (b) Testing set**. The prediction results of all the proteins in the datasets were calculated and averaged. The precisions of random prediction are 15% and 12.6% for the training and testing sets, respectively.

As shown in Table 2, all individual terms showed only slightly better prediction than a random prediction and the performance varies with the selected protein datasets. Combination of multiple features to increase the prediction accuracy is a challenge. Recently, Sweredoski & Baldi concluded that non-linear methods such as SVMs, ANNs, and Gaussian Mixture Models did not achieve higher performance than a linear combination [36]. Here, we found that the precision of most terms does not increase for both training and testing sets as the number of predicted residues decreases except for conservation score (Fig 1). Selection of top scored residues by linear combination has no advantage over the consensus prediction of several terms. In fact, when the contribution of the six terms was considered to be equal (as an example) and the sum of the six scores was used for re-ranking, we obtained an average AUC of 0.603 for the training set. When the residues, which were predicted as interface residues by 5 out of the 6 terms, were selected as the final prediction, the AUC value was 0.614 for the training set.

The precision of the conservation score always increases for both the training and testing sets as the number of predicted residues decreases. The residues scored above the cutoff value by only conservation were also considered as interface residues. Furthermore, when the predicted residues with the single term are less than 28% of total surface residues, none of interface residues are predicted by consensus scoring for at least one training protein and interface residues are only predicted by conservation score. We tried the cutoff value of 5%, 10%, 15%, and 20%, and the AUC values were 0.619, 0.622, 0.626, and 0.618 respectively. The cutoff value (15%) yielding the best AUC value was selected.

All the residues in the surface patch contributed equally to the patch score in the above predictions and only the central residues of the top scored patches were selected. In order to optimize the patch score with respect to the position of residues in the patch, the score of each patch residue was considered assuming an exponential decrease of the weight with the distance between patch residues and the central residue. The decay constant *T *in equation (3) was allowed to take the values 4, 8, 12, and 16. The corresponding AUC values were 0.622, 0.633, 0.635, and 0.629, respectively, for the training set. The *T *value of 12 yielding the best AUC value was selected for applications on the test set. Table 3 lists the results for the training set.

**Table 3:** Prediction results for the training set with 6 combined terms

***Complex***	***Unbound***	***No. of Surface Residues***	***No. of Interface Residues***	***Sensitivity*^*a*^**	***Precision*^*a*^**	***Specificity*^*a*^**	***AUC***
1AHW_AB:C	1TFH_A	173	25	0.360	0.153	0.662	0.481
1BVK_DE:F	3LZT_	98	17	0.765	0.361	0.716	0.835
1DQJ_AB:C	3LZT_	98	20	0.300	0.182	0.654	0.534
1E6J_HL:P	1A43_	63	13	0.462	0.353	0.780	0.585
1JPS_HL:T	1TFH_B	155	25	0.360	0.170	0.662	0.517
1 MLC_AB:E	3LZT_	98	16	0.562	0.250	0.671	0.636
1VFB_AB:C	8LYZ_	107	18	0.833	0.385	0.730	0.833
1WEJ_HL:F	1HRC_	95	13	0.462	0.188	0.683	0.649
2VIS_AB:C	2VIU_A	247	20	0.900	0.281	0.797	0.901
1BJ1_HLJK:VW^b^	2VPF_GH	160	35	0.600	0.412	0.760	0.705
1FSK_BC:A	1BV1_	145	19	0.526	0.233	0.738	0.587
1I9R_HL:ABC^b^	1ALY_ABC	320	65	0.508	0.292	0.686	0.687
1IQD_AB:C	1D7P_M	127	17	0.765	0.361	0.791	0.848
1K4C_AB:C	1JVM_A	88	16	0.500	0.258	0.681	0.647
1KXQ_H:A	1PPI_	341	30	0.600	0.148	0.666	0.637
1NCA_HL:N	7NN9_	263	27	0.556	0.163	0.674	0.684
1NSN_HL:S	1KDC_	106	23	0.174	0.114	0.627	0.454
1QFW_HL:AB	1HRP_AB	170	17	0.235	0.071	0.660	0.484
1QFW_IM:AB	1HRP_AB	170	17	0.706	0.214	0.712	0.738
2JEL_HL:P	1POH_	68	18	0.167	0.158	0.680	0.498
1BGX_HL:T	1CMW_A	646	66	0.394	0.124	0.683	0.521
2HMI_CD:AB	1S6P_AB	810	14	0.429	0.024	0.697	0.518
Mean		207	24.1	50.7	0.222	0.7	0.635

^a ^Sensitivity, precision, and specificity were recorded when 55% of surface residues were predicted as interface residues by the single term. We chose the parameter (55%) so that the sensitivity was about 50% in the consensus prediction. ^b^Multiple binding sites.

### Independent test

**Figure 2:**
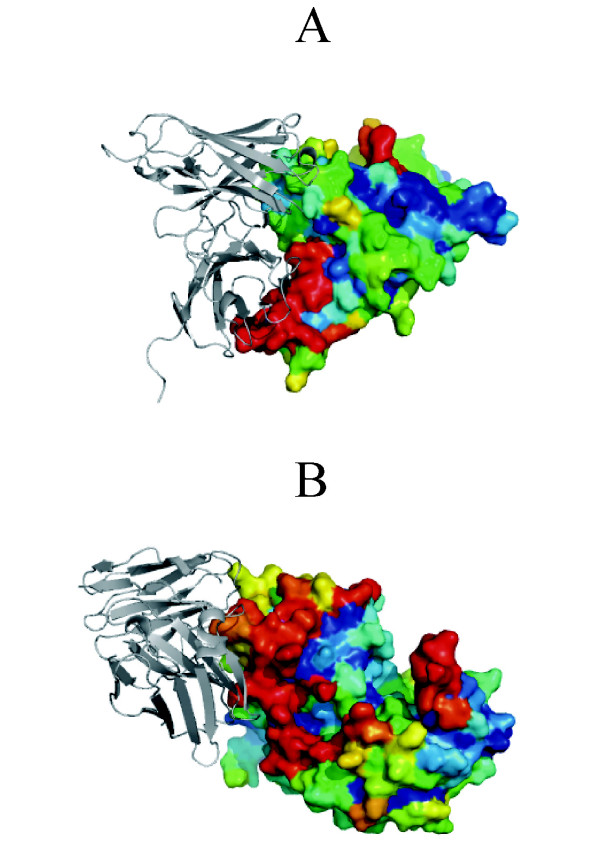
**Two successful examples of antibody binding site prediction (a) SARS spike protein receptor binding domain **(2ghv); **(b) Cockroach allergen Bla g 2 **(1yg9). The antibodies were colored in grey. The surface residues of antigens were colored according to predicted possibility to be an epitope residue (from red to blue in decreasing order) and the core residues were colored in blue.

The average AUC value was 0.616 for the testing set when the residues predicted as interface residues by 5 of the 6 terms were selected in the consensus prediction. The AUC value increased to 0.621 when the residues top scored by conservation were included in the prediction (cutoff value = 15%) and further increased to 0.632 (Table 4) if non-uniform averaged patch scores were used (*T *= 12). The prediction accuracy was very close to that for the training set (0.635). Fig. 2 shows two successful predictions. The real antibody binding sites overlapped with the largest cluster of red colored residues which correspond to the predicted antigenic epitope residues.

**Table 4:** Prediction results for the testing set

***Complex***	***Unbound***	***No. of Surface Residues***	***No. of Interface Residues***	***Sensitivity*^*a*^**	***Precision*^*a*^**	***Specificity*^*a*^**	***AUC***
2ARJ_HL:Q	1NEZ_G	99	18	0.278	0.227	0.790	0.604
2BDN_HL:A	1DOK_A	63	13	0.154	0.095	0.620	0.281
2FD6_HL:U	1YWH_A	225	14	0.500	0.089	0.659	0.617
2GHW_B:A	2GHV_E	148	27	0.519	0.311	0.744	0.727
2H9G_AB:R	1D4V_A	108	18	0.556	0.286	0.722	0.724
2J6E_IMHL:AB^b^	2DTQ_AB	336	41	0.512	0.202	0.719	0.614
2NR6_CD:A	1YG9_A	233	19	0.947	0.234	0.724	0.870
2NYY_CD:A	2VUA_A	321	24	0.750	0.164	0.690	0.810
2P45_B:A	1KF2_A	104	13	0.154	0.057	0.637	0.553
2Q8B_HL:A	1Z40_A	228	25	0.440	0.147	0.685	0.645
2QQN_HL:A	1KEX_A	118	11	0.636	0.200	0.738	0.737
2R29_HL:A	1OK8_A	317	20	0.300	0.054	0.646	0.567
2R56_HL:A	1GX9_A	131	22	0.091	0.053	0.670	0.409
2UZI_HL:R	2EVW_X	132	21	0.286	0.171	0.739	0.505
3BN9_CD:B	1EAX_A	181	32	0.531	0.279	0.705	0.581
3BQU_CD:AB	2F5A_HL	336	12	1.000	0.098	0.660	0.914
3D85_AB:C	3D87_A	141	19	0.474	0.180	0.664	0.591
Mean		189	20.5	47.8%	16.7	69.5%	0.632

^a ^Sensitivity, precision, and specificity were recorded when 55% of surface residues were predicted as interface residues by the single term. ^b^Multiple binding sites.

### Comparison with other epitope prediction methods

In this study, we investigated residue antibody binding site propensity based on atomic solvent accessibility for 20 amino acids. The AUC values for training and testing sets were 0.637 and 0.577, respectively, when the single term of propensity score were used for prediction. Currently, two other algorithms using multiple features for antibody binding site prediction are available, DiscoTope[20] and PEPITO[36]. These methods used similar antibody binding site propensity scores at residue level. We also tried using the propensity score of DiscoTope [20] for comparative predictions. The AUC values were 0.587 and 0.551 for our training and testing sets, respectively. Propensity score based on atomic solvent accessibility has a slightly better performance than the propensity score of residue level for both datasets.

We compared our consensus algorithm with the recently updated version of DiscoTope[20] and PEPITO[36], DiscoTope 1.2  and BEpro . The computation was conducted on the websites for each method between December 2008 and January 2009. As shown in Table 5, all of the algorithms have similar prediction for the training set while our algorithm showed a better prediction (AUC = 0.632) for the unbound structures of testing set than DiscoTope (0.589) and BEpro (0.598). It should be noted that all the PDB files in the testing set were released very recently so these structures were not part of the training set for the two published methods and could serve as independent testing cases for other algorithms as well. In the original paper an AUC value of 0.71 was reported for DiscoTope averaged over five evaluation sets used for cross validation [20]. However, a considerable smaller AUC value for DiscoTope of 0.566 for 30 targets out of 59 representative antigen-antibody complexes, which were compiled by Ponomarenko & Bourne and not used for training DiscoTope was reported in a recent study [25]. The prediction accuracy of recently released DiscoTope 1.2 was slightly improved compared with the original DiscoTope in the two independent tests.

**Table 5:** Comparison with other algorithms

***Methods***	***Training set***		***Testing set***	
	**Bound**	**Unbound**	**Bound**	**Unbound**
DiscoTope1.2	0.63	0.628	0.6	0.589
BEpro	0.645	0.639	0.617	0.598
Our algorithm	0.628	0.635	0.603	0.632

Unlike Discotop1.2 and BEpro, our algorithm has lower prediction accuracy for the bound structures than the unbound structures due to the inclusion of the side chain energy score. The interface residues of bound antigen are buried in the complex and usually have a lower temperature factor than other surface residues. In the bound forms these side chains have systematically lower energies than in the unbound form which in our algorithm contributes unfavorably to the score [27]. Predictions with the side chain energy score as single term yielded AUC values of training and testing sets of 0.555 and 0.569, respectively, for unbound structures and 0.532 and 0.521 for bound structures, respectively.

## Conclusion

An important conclusion of the present study is that antibody binding site prediction is more difficult than prediction of other protein binding regions. A combination of multiple surface features which allows relatively accurate prediction of protein binding sites in general shows poorer performance in case of antibody binding site prediction. An important issue is also that a given protein usually contains not only one but several putative antibody binding sites. Usually an antibody-antigen complex structure indicates only one of these possible antigenic epitopes. In addition, care must be taken in evaluating prediction methods when a relatively small number of antibody-antigen complexes were used as the testing set. The prediction algorithm may work reasonably well on one testing set but could show poorer prediction accuracy on new targets due to different interface properties. More training proteins are required for developing new prediction algorithms in the future. Nevertheless, the study demonstrated that consensus scoring of widely used features for binding site prediction showed a better performance than any single term for the independent test set. The prediction accuracy was improved further by utilizing residue epitope propensity based on atomic solvent accessibility. However, a detailed comparison with other published methods indicated that overall the performance of our combined approach is similar to existing methods.

## Availability

The EPCES program is available upon request. A web-based EPCES application is available at: .

## Authors' contributions

SL designed the study, implemented the algorithm and drafted the manuscript. DZ and CZ helped prepare the data, write the software and draft the manuscript. MZ supervised the study. All authors read and approved the final manuscript.
